# Protecting genomic data analytics in the cloud: state of the art and opportunities

**DOI:** 10.1186/s12920-016-0224-3

**Published:** 2016-10-13

**Authors:** Haixu Tang, Xiaoqian Jiang, Xiaofeng Wang, Shuang Wang, Heidi Sofia, Dov Fox, Kristin Lauter, Bradley Malin, Amalio Telenti, Li Xiong, Lucila Ohno-Machado

**Affiliations:** 1 School of Informatics and Computing, Indiana University, Bloomington, IN USA; 2 Department of Biomedical Informatics, University of California San Diego, La Jolla, CA USA; 3 National Human Genome Research Institute, Rockville, MD USA; 4 School of Law, University of San Diego, San Diego, CA USA; 5 Microsoft Research, Redmond, WA USA; 6 Department of Biomedical Informatics, School of Medicine, Vanderbilt University, Nashville, TN USA; 7 The J. Craig Venter Institute, La Jolla, CA USA; 8 Department of Mathematics and Computer Science, Emory University, Atlanta, GA USA

## Abstract

The outsourcing of genomic data into public cloud computing settings raises concerns over privacy and security. Significant advancements in secure computation methods have emerged over the past several years, but such techniques need to be rigorously evaluated for their ability to support the analysis of human genomic data in an efficient and cost-effective manner. With respect to public cloud environments, there are concerns about the inadvertent exposure of human genomic data to unauthorized users. In analyses involving multiple institutions, there is additional concern about data being used beyond agreed research scope and being prcoessed in untrused computational environments, which may not satisfy institutional policies. To systematically investigate these issues, the NIH-funded National Center for Biomedical Computing iDASH (integrating Data for Analysis, ‘anonymization’ and SHaring) hosted the second Critical Assessment of Data Privacy and Protection competition to assess the capacity of cryptographic technologies for protecting computation over human genomes in the cloud and promoting cross-institutional collaboration. Data scientists were challenged to design and engineer practical algorithms for *secure outsourcing* of genome computation tasks in working software, whereby analyses are performed only on encrypted data. They were also challenged to develop approaches to enable *secure collaboration* on data from genomic studies generated by multiple organizations (e.g., medical centers) to jointly compute aggregate statistics without sharing individual-level records. The results of the competition indicated that secure computation techniques can enable comparative analysis of human genomes, but greater efficiency (in terms of compute time and memory utilization) are needed before they are sufficiently practical for real world environments.

**Electronic supplementary material:**

The online version of this article (doi:10.1186/s12920-016-0224-3) contains supplementary material, which is available to authorized users.

## Author summary

Advancement of technology significantly reduces the price of obtaining whole genome sequencing (WGS) data and makes personalized genome analysis more affordable. The increasing availability of human genomic data is accompanied with increasing privacy concerns, such that the inappropriate disclosure of such data might put individuals at risk. In this paper, we present the recent findings of novel genomic data protection methods through a community-wide open competition to address the emerging privacy challenges. The goal of the competition is to bridge the gap between the biomedical informatics, data privacy, and security communities.

## Introduction

Advances in high throughput technologies have made it increasingly affordable to sequence the human genome in various settings, ranging from biomedical research and healthcare. Massive collection of human genomic data [1], together with the advancement of analysis techniques, may enable more effective clinical diagnosis, as well as the discovery of new treatments. Given such potential, there are numerous initiatives that have been established, the most recent of which is the Precision Medicine Initiative, which will aim at studying the combined genotypes and phenotypes of at least one million volunteers [2].

At the same time, the collection of such a large quantity of data leads to new challenges. The latest version of the NIH Genome Data Sharing (GDS) policy (http://grants.nih.gov/grants/guide/notice-files/NOT-OD-15-086.html) allows users to store and analyze human genomic data downloaded from NIH repositories in cloud environments, as they provide solutions to the storage and computation limitations researchers often face when handling large genomic datasets. However, to take advantage of such environments, it was stated in the *NIH Security Best Practices for Controlled-Access Data Subject to the NIH Genomic Data Sharing (GDS) Policy* (http://grants.nih.gov/grants/guide/notice-files/NOT-OD-15-086.html) that the scientists and their institutions are responsible for ensuring the security of human genomic data (i.e., it is not a responsibility of the cloud service provider). Whatever care biomedical researchers might afford to potentially sensitive health information cannot replace the formal procedures required to secure such data and keep it protected.

In the United States, research use of human genomic data will likely soon be governed by the so-called Common Rule, if it wasn’t already. That Rule does not (for now) expressly define human genomic data. But revisions to that federal policy due out later this year are expected to include genomic data within its human subjects protections. The NIH GDS operates to protect research subjects’ personal information by recoding or removing 18 explicit identifiers (e.g., personal names or study IDs). The GDS adopts these criteria for defining de-identified data in clinical settings from the safe harbor model provided by the Health Insurance Portability and Accountability Act of 1996 (HIPAA) [3]. Human genomic data, linked or otherwise, could accordingly be considered “de-identified” for research uses.

Additionally, there is concern because genomic data, even when devoid of other explicit identifiers, can, at times, be re-identified by invoking various methods (e.g., side-channel leaks [4], completion attacks [5], and genealogical triangulation [6]). As recent studies have demonstrated, the human genome can, at times, communicate information about an individual’s appearance and/or heritage, as well as their predisposition to disease [5–10]. The human genome may even provide evidence of criminal culpability, such as being present at a crime scene [11], which makes the responsible use of such information especially important in certain legal situations. Even more concerning is that, though the privacy risk due to the known re-identification techniques is largely limited, the privacy risk may increase over time with the further accumulation of knowledge about human genetics and the development of new re-identification techniques. Once genomic data is made public, it remains permanently public. Finally, genomic data communicates information about people beyond the individual from whom the data was collected (e.g., parents, siblings, children, and cousins), and thus contributes to the risks for others.

In the past few years, there has been a growing interest in developing effective, secure and privacy-preserving methodologies to analyze sensitive genomic data. Recent reviews [5, 12] have discussed relevant techniques. These solutions seek to make data usable for medical research (and clinical applications) while effectively preventing the disclosure of private information. However, it remains unclear how well these techniques perform when applied to large quantities of genomic data. This is problematic because most cryptographic protocols are beset by limited scalability when they are applied to whole genome association studies, and cryptography solutions have yet to completely solve the problem of how to secure data-sharing for analysis. There has been no direct comparison of the range of methods in practical scenarios (e.g., using common benchmarks). As a result, it is difficult for biomedical researchers to understand what has been achieved and what remains to be done given the status of current technology.

To better understand the limits of the state-of-the-art in protecting computation over genomic data with cryptographic techniques, we organized the second Critical Assessment of Data Privacy and Protection (CADPP) workshop as a community effort to evaluate the effectiveness of relevant methodologies. To set the competition in a realisitic context of collaborations, we focus on an honest but curious adversary model, in which a semi-honest cloud server corrupted by an adversary returns the expected result following the computational task but keeps exploring the data (as well as the computation process) in an attempt to infer sensitive information. Our goal is to mitigate the privacy risk of using a public commercial cloud, especially in the cases where the security of the cloud is achieved at a high cost.

The findings of the competition (summarized in Table 1) are significant, providing insights into where the technologies stand and what needs to be accomplished to move them closer to serving the biomedical researchers and clinical practitioners. In particular, the competition was designed to focus on two practical applications: (1) *secure outsourcing* and (2) *secure collaboration*. For the first application, we assume that the data owner has limited resources and wants to securely outsource data storage and computation to a third-party cloud service. For the second application, multiple data owners (e.g., two or more medical institutions) want to analyze genomic data jointly without disclosing their own data. Based on these application scenarios, we devised two tasks including both genome-wide association studies (GWAS) and whole genome sequence comparison for each challenge, based on publicly available human genomic data from the International HapMap project [13] and the Personal Genome Project (PGP) [14]. The workshop for the competition was attended by a broad community, with 11 participating teams (Additional file 1: Figure S1).

**Table 1:** Significant findings of the second Critical Assessment of Data Privacy and Protection (CADDP) competition

1. *Certain important genome analysis tasks can already be protected on a large scale*. As a prominent example, it was found that the state-of-the-art privacy-enhancing technologies (PET) can already support the calculation of Hamming distance (a widely used distance measure for genomic sequences) across genomes with 100,000 bases in a few minutes, even when the genomic data are fully encrypted or the computation is performed across two geographically distributed institutions. These indicate that it is realistic to use cloud or secure multiparty computation for certain genomic data analysis tasks, even when the cloud or each party are not fully trusted, while still maintaining sufficient protection of patient privacy and scalability of the computation.
*2. Gaps remain for certain classes of biomedical computations.* When it comes to more complicated computations (e.g., association tests for a GWAS), analyzing encrypted data on a commercial cloud involves a large computational and communication burden.
*3. Narrowing the gap between data usefulness and privacy protection requires a joint effort from the biomedical and security communities.* Cryptographers and computer scientists need to collaborate with biomedical researchers to move PET techniques closer to practice. We found that an approximation of the Edit distance computation on human genomes is very effective, significantly simplifying computation and allowing it to be calculated, securely, on a large scale.

## Material and methods

### Secure computation technologies

For the first application, *secure outsourcing of computation*, we challenged participating teams to safeguard genomic data storage and computation by using homomorphic encryption (HME) techniques [15]. Homomorphic encryption allows computation to be carried out on ciphertexts, thus generating an encrypted result, which, when decrypted, matches the result of operations performed on the plaintext [16]. This is a general technique, which allows data owners to encrypt their data and analyze it using a cloud computing service anytime in the future.

In detail, there are three types of homomorphic encryption techniques: (1) partial HME is specialized on a single operation (either addition or multiplication) [17], (2) full HME that allows both operations but less efficiently [18–21], and (3) levelled HME that allows both operations for a limited number of iterations [22]. Partial HME techniques, such as unpadded RSA: $E(x)={x}^e\; mod\;m$ (modulus $m$ and exponent $e$ as the public key), are very efficient: $E\left({x}_1\right)E\left({x}_2\right)={x}_1^e{x}_2^e\; mod\;m={\left({x}_1{x}_2\right)}^e\; mod\;m=E\left({x}_1{x}_2\right),$ but they cannot be combined with another operation, such as addition. Since more complex primitives (e.g., those used in human genome computation) involve multiple operations, partial HME has limitations. A full HME system can conduct both operations and can implement any function in theory; however, it is very computation and storage intensive. Levelled HME requires a pre-specification of the number of operations: it is a compromise between the above two techniques and it offers reasonable efficiency. There have been considerable advances in using HME to protect genome privacy [23, 24].

For the second application, *secure collaboration*, we challenged participating teams to implement secure multiparty computation (SMC) solutions that are customized for data analysis tasks [25]. In this competition, we focused on a two-party scenario, where two participating parties (e.g., medical institutions) aim to jointly compute a function over their inputs, while keeping these inputs private. Each party can perform a certain computation locally on the controlled-access (private) data and exchanges only intermediate results to synthesize a global output that can be shared by both parties. There should be no additional information leakage during the computation. SMC protocols often require synchronization and involve a large amount of peer-to-peer communication.

In the past few years, secure protocols have been developed for various genomic applications. HME based GWAS, risk prediction, and sequence comparison have been explored by Wang et al. [26, 27], Lauter et al. [28, 29], Cheon et al. [30], and Ayday et al. [31, 32]. Danezis and Cristofaro [33], Djamiko et al. [34], Verle et al. [35], and Lu et al. [35] developed SMC-based approach for secure function evalution and statistical tests. Kantarcioglu et al. [36] and Mohammed et al. developed cryptographic approaches to share and query genomic sequences [36]. Huang et al. developed a new tool GenoGuard based on honey encryption, which produces a plausible-looking yet incorrect plaintext to protect against brute-force attacks [37]. However, there has been no systematic comparison of different methods on a common human genome benchmark dataset.

### Challenge design

We organized the competition as two applications: (1) secure outsourcing of human genome computation using HME techniques (Application 1), and (2) secure collaboration on human genome computation by using SMC techniques (Application 2). For each challenge, we devised two tasks: (1) secure GWAS, and (2) secure genome sequence comparison. To evaluate the performance of the submitted algorithms, we provided template virtual machines (VMs) for the participating teams to train and configure their algorithms. The same machines were subsequently relied upon after submission in the final evaluation step. For the secure collaboration challenge, two VMs were provided and were hosted at UC San Diego and Indiana University, respectively.

In the GWAS task, we created case and control groups, using their real genotypes in the challenge. The 200 cases were acquired from the PGP with missing genotype values filled by using fastPHASE [38]. Another 200 controls were simulated based on the haplotypes of 174 individuals from the HapMap Project. Each participating team (for both applications) was given the genotypes of the case and control groups over 311 SNP sites (for training purposes). They were then asked to implement an algorithm to conduct a case-control association test by using ${\chi}^2$ -test statistics on these SNP sites. The submitted algorithms were also evaluated on another genomic dataset (on the same case/control genomes) over 610 additional SNP sites.

For the secure outsourcing application, each participating team was required to develop an HME-based protocol to encrypt the input datasets and calculate minor allele frequencies (MAFs) and ${\chi}^2$ -test statistics on a semi-honest server. The protocol should return the encrypted results and only the data owner with the private key could decrypt the result. The data owner could be involved in the final calculation (e.g., compare the AF and calculate the ${\chi}^2$ -test statistics from intermediary results), but the goal was to maximize the computational outsourcing and minimize the interaction and communication. For the secure collaboration application, the input case and control dataset was horizontally partitioned into two sub-datasets (100 cases and controls in each sub-dataset) distributed to two institutions (i.e., the two VMs at UC San Diego and Indiana University), where each institution hosted a single sub-dataset that could not be exchanged. Each participating team was required to develop a distributed cryptographic protocol to securely compute MAFs and ${\chi}^2$ -test statistics for each given SNP site in a two-party scenario.

For the sequence comparison task, two individual genomes (i.e., hu604D39 with 4,542,542 variations and hu661AD0 with 4,368,847 variations compared to the reference human genome) were taken from PGP. Given the genome sequences (in variant call format, or VCF) from these two PGP individuals, a subset of variation sites were randomly selected to form the input data of different sizes (5 K and 100 K) for training, whereas distinct subsets of variation sites (of size 5 K, 10 K, and 100 K) were used for evaluation. For the secure outsourcing application, each team was provided the two genome sequences (in VCF) and asked to develop a HME-based cryptographic protocol to outsource the storage and computation for calculating Hamming distance and edit (Levenshtein) distance between the two genome sequences. For the secure collaboration application, the two genome sequences (in VCF) were distributed to two institutions (i.e., the VMs at UCSD and Indiana), where each institution hosted a single genome and could not exchange genomes. Each team was required to develop a distributed cryptographic protocol to securely compute the Hamming distance and edit distance between two human genomes across institutions.

#### Approximate edit distance calculation

The edit distance computation (i.e., following the dynamic programming algorithm) is known to be expensive when using secure computation protocols. State-of-the-art algorithms compute the distance between two sequences of lengths only 2 K and 10 K, at a cost of more than 3.5 h and 38 GB of network traffic [39]. To address this issue, we devised an approximation algorithm to compute the edit distance between two human genomic sequences. Briefly, given two human genome sequences represented by their variations from the reference genome sequences in the variant call format (VCF), their edit distance is approximated by the size of the set difference between these two variation sets. Note that the VCF representation of each individual genome can be computed privately at each institution without collaborating with the other party; this can be achieved by variation calling algorithms such as GATK [40].

We applied the algorithm to the comparison of 20 pairs of human genomic segments of about 5000 nucleotides and found that it performed well in practice. For 18 of the cases, it reported the exact true edit distance, in 1 case, it reported an approximate distance 1 (3.7 %) higher than the true distance (28 vs. 27), and in the final case, the approximate distance significantly (6.0 %) deviated from the true distance (48 vs. 51). This algorithm was recommended to all participating teams for the genome comparison tasks of computing edit distance between two human genome sequences. We refer the reader to the competition website (http://www.humangenomeprivacy.org/2015/competition-tasks.html) or a recent paper [41] for further details about the approximation algorithm.

### Submission and evaluation

Each participating team was asked to submit a suite of software programs with an implementation of their algorithms (either binary executable files or source code), precompiled on supplied preset VMs. The performance of the algorithms was evaluated by the organizers on holdout test datasets (as described above). The following criteria were considered in ranking the participating teams.

- Accuracy: Each solution should achieve the minimum required accuracy.
- Security: Each solution should fulfill the minimum-security protection standard (i.e., under the semi-honest attack model with at least 80-bit security level). 80-bit security level indicates that it requires a computer to perform, on average, approximately 2^80^ operations to break the encryption. A semi-honest attack model was defined by the assumption that each party followed the protocol while they could use any observed (including intermediate) computation to try and discern sensitive information about the data.
- Complexity: The execution time of each solution was measured under the same or comparable software and hardware setups. The evaluation included 1) the running time for encryption, 2) the computation over encrypted data, and 2) and decryption of the solution.
- Storage: Since data encryption increases the size of the data, we measured the storage efficiency of each solution after applying the proposed cryptographic protocols.
- Communication Cost: Cryptographic protocols involve the communication of encrypted data between computational nodes. We measured the communication cost in terms of network bandwidth consumption.

## Results

A total of 11 teams participated in the competition (5 for the first application and 7 for the second application with one team participating in both). Based on the performance, we awarded six winners, three for each application based on different tasks in Table 2. Tasks 1.1 and 2.1 were designed for computing MAF and ${\chi}^2$ -test statistics for secure outsourcing and collaboration, respectively. Tasks 1.2 and 2.2 address whole genome comparison for secure outsourcing and collaboration, respectively.

**Table 2:** A summary of tasks, participating teams, and winners of the challenge

	GWAS	Whole genome comparison
Application 1: Secure outsourcing	Task 1.1 – MAF & ${\chi}^2$ (Winner: Stanford/MIT)	Task 1.2 (a) – Hamming distance (Winner: IBM)
		Task 1.2 (b) – Approximate Edit distance (Winner: Microsoft)
	Participating teams: IBM; Stanford/MIT; Microsoft; UC Irvine (UCI); University of Tsukuba	
Application 2: Secure collaboration	Task 2.1– MAF & ${\chi}^2$ (Winner: U of Maryland)	Task 2.2 (a) – Hamming distance (Winner: University of Virginia)
		Task 2.2 (b) – Approximate Edit distance (Winner: UC Irvine)
	Participating teams: Syracuse University (SU); University of Maryland (UMD); University of Notre Dame (UND); University of Virginia (UV); UC Irvine (UCI); Cybernetica AS (CAS); The Alexandra Institute (AI)	

The details of performance are summarized in Tables 3 and 4 for each task. For the first application, secure outsourcing, we compared accuracy, time, and memory. All participating teams achieved accurate results for MAF and ${\chi}^2$ -test statistics, such that we only compared time and memory in Task 1.1. For Task 1.2, we separated the comparison for Hamming distance and approximate edit distance. Among teams that participated in the HME application, IBM Research, Microsoft Research, and University of Tsukuba used HELib (https://github.com/shaih/HElib), which is an open source library for full HME developed by the IBM team. UCI and Standford/MIT used a partial HME scheme, Paillier encryption, to encode and compute MAF and ${\chi}^2$ -test statistics.

**Table 3:** A summary of the results for the secure outsourcing tasks

Task 1.1		MAF				${\chi}^2$				
		311 SNPs		610 SNPs		311 SNPs		610 SNPs		
		Time	Memory	Time	Memory	Time	Memory	Time	Memory	
	Microsoft	17.44 s	130.48 MB	26.31 s	247.30 MB	16.88 s	118.08 MB	27.11 s	234.73 MB	
	UCI^a^	0.589 s	3.320 MB	0.886 s	3.320 MB	0.659 s	3.320 MB	0.871 s	3.320 MB	
	**Stanford/MIT**	**1.069 s**	**8.0 MB**	**1.847 s**	**13.0 MB**	**1.069 s**	**8.0 MB**	**1.847 s**	**13.0 MB**	
	U of Tsukuba	55.20s	31.81 MB	112.32 s	32.67 MB	55.21 s	31.81 MB	112.32 s	32.67 MB	
Task 1.2 (a)		5 k			10 k			100 k		
		Accuracy	Time	Memory	Accuracy	Time	Memory	Accuracy	Time	Memory
	Plaintext data	3099	0.076 s	1.64 MB	3306	0.118 s	2.43 MB	134252	134252	13.52 MB
	**IBM**	**3099**	**79.4 s**	**1.416 GB**	**3306**	**86.8 s**	**1.419 GB**	**134260**	**134260**	**2.168 G**
	Microsoft	3099	44.664 s	513.7 MB	3306	80.031 s	720.5 MB			
	Stanford/MIT	3082	20m37s	2.77 GB	3275	36m27s	4.03 GB	132703	132703	7.50 GB
Task 1.2 (b)		5 k			10 k			100 k		
		Accuracy	Time	Memory	Accuracy	Time	Memory	Accuracy	Time	Memory
	Plaintext data	9089	0.106 s	2.45 MB	16667	0.144 s	2.53 MB	191986	1.528 s	25.8 MB
	IBM^b^	5328	91.7 s	1.42 GB	8318	106.3 s	1.45 GB	153266	555.2 s	2.29 GB
	**Microsoft**	**9089**	**91.09 s**	**701 MB**	**16665**	**181.92 s**	**1.29 GB**			

^a^The algorithm encrypts local counts instead of input data for secure data outsourcing, and was disqualified in the competition

^b^An approximate algorithm (with about 22 % error), which was not considered in the competition

Winners are in boldface

**Table 4:** A summary of the results for the secure collaboration tasks\

Task 2.1		311 SNPs ${\chi}^2$ -statistics							610 SNPs ${\chi}^2$ -statistics						
		Time (s)	Memory (KB)			Communication (MB)			Time (s)	Memory (KB)			Communication (MB)		
			VM1	VM2	VM3	VM1	VM2	VM3		VM1	VM2	VM3	VM1	VM2	VM3
	Baseline	92	1.2	1.4		0.7	35.0		187	1.2	1.4		1.4	70.0	
	UV	32	3.3	5.3		1.9	163.0		59	6.9	9.7		3.6	309.3	
	UND	15	25.1	25.1	25.0	4.0	3.8	3.8	23	36.2	49.8	36.0	7.9	7.4	7.2
	SU	14	173	162		4942	45.6		54	187	175		9645.7	93.0	
	**UMD**	**13**	**63.5**	**58.1**		**0.8**	**46.2**		**20**	**71.3**	**64.6**		**1.6**	**90.7**	
	CAS	60	0.1	0.1	0.1	0.007	0.007	0.007	57	0.1	0.1	0.1	0.007	0.007	0.007
Task 2.2		Hamming distance (~100 K)							Approximate Edit distance (~100 K)						
		Time (s)	Memory (KB)			Communication (MB)			Time (s)	Memory (KB)			Communication (MB)		
			VM1	VM2	VM3	VM1	VM2	VM3		VM1	VM2	VM3	VM1	VM2	VM3
	Baseline								254	290	292		92.0	5595.0	
	UMD	604	1260	1252		63.4	2973.3		>20 h						
	UMD (BF)^a^	83	0.1	0.1		19.8	150.8		233	145	125		50.2	424.5	
	**UCI**	788	0.4	0.4		28.8	24.4		**998**	**434**	**398**		**39.1**	**32.7**	
	CAS^b^	128	0.4	0.4	0.4	0.1	0.1	0.1							
	**UV**	**553**	**0.3**	**0.3**		**156.5**	**9672.9**								
	UND	5077	3044	3048	3048	4118.5	3361.7	3167.3							
	AI								>20 h						

^a^An approximate algorithm (with about 0.8 % error) based on Bloom filters, which was not considered in the competition

^b^The algorithm involves intensive computation on the third server, and thus was not considered in the competition

Winners are in boldface

The algorithms developed in this competition demonstrate the feasibility of conducting common genome data analysis tasks in a secure and privacy-preserving manner. With 80 bits of security, it is possible to transfer and analyze personal genome data, e.g., on Amazon Web Service (AWS), in less than 15 s for calculating minor allele frequency and chi-squared statistics of 611 SNPs). Larger scale genome similarity is also feasible (less than 8 min for calculating Hamming distance for 100 k SNPs and about 3 min for calculating the Edit distance for 10 k SNPs). For the SMC, the best solution needs 20 s for computing *χ* ^2^ statistics of 610 SNPs. The best solutions for calculating Hamming and edit distances over 100 K SNPs are about 10 and 17 min, respectively.

In addition, five teams extended their work and published them in a special issue.^1^ Three of them are about secure outsourcing using homomorphic encryption. Lu et al. [42] showed that genome wide association test (GWAS) on 1 million SNPs can be computed in less than 11 h. Kim and Lauter [43] demonstrated that the Hamming and Edit distances for sequences of 10 k length can be computed in less than 60 and 120 s, respectively. Zhang et al. [44] demonstrated comparable results. Two other articles are about secure multiparty computation, which supports decentralized data analysis without the need of a coordinator. Constable et al. [45] described a solution for two parties to securely compute minor allele frequency (MAF) and *χ* ^2^ statistics for 9330 SNPs in 9.4 and 22.22 min, respectively. Zhang et al. [46] showed a secret sharing model (slightly utilizing a third party) has very good efficiency (MAF and Chi-squared statistics are calculated in 2.5 and 77 s, respectively).

Despite these encouraging results, the competition also revealed a number of limitations of current techniques. For example, existing cryptographic techniques showed huge overhead in storage and communication: HME used up to several gigabyte of memory, while SMC needed to transmit multi-gigabytes of data across the Internet, for analyzing genomic sequences of length 100 K, posing challenges for their scalability. There is room for improvement, but we also need to apply the right technology to the right problem (e.g., SMC would be more appropriate than HME for researchers who want to compare small regions across a large number of genomes).

## Discussion

The outcomes of the competition highlighted progress made in cryptographic technologies: we can already protect important computations such as edit distance and Hamming distance calculations on encrypted genomic data and across organizations at a scale close to practical use. This indicates that it is possible to move genomic data analysis to the cloud, even when the cloud is not fully trusted, while still maintaining sufficient protection of patient privacy and scalability of the computation. The findings further demonstrate that cryptographic techiniques can offer the biomedical communities powerful tools to safeguard data while utilizing the computing power provided by modern cloud computing platforms.

At the same time, we show that complicated analyses (e.g., association tests) call for new technologies to achieve efficient secure computation on cloud platforms. Potential ways to move forward include the better design of cryptographic primitives, which the security community is aggressively pushing forward (e.g., the performance of full HME has been improved by several orders of magnitude in the past few years), as well as tailoring the primitives to the unique features of genomic data. A notable example is a computation partition [29], which offloads some computation to the local system of the data owner to simplify the work that needs to be performed in the public cloud.

Most importantly, the competition shows that the protection of genome privacy is truly an interdisciplinary area: progress is being made on both security and genomic fronts. In particular, secure computation of edit distance had been studied in the security community [27, 30], but state-of-the-art techniques remain insufficient for supporting the analysis on a scale practical for biomedical research. Yet by leveraging characteristics of the human genome, we found that an approximation of the computation on human genomes is very effective, which significantly simplifies computation and allows the edit distance to be calculated securely and on a large scale. We believe that similar efforts can be successful for other genomic data analysis algorithms. More importantly, these observations point to the need for closer collaborations between biomedical and security researchers and for fostering this emerging interdisciplinary research area.

Mounting risks to human genome privacy motivate the development of efficient and scalable cryptographic techniques, which should be customized for practical data analysis applications. The 2015 CADPP workshop brought together the security and biomedical informatics communities to join forces in closing the gaps, and proposing novel and practical solutions. We learned the capacity and limitation of state-of-the-art algorithms and discovered that a careful tweaking of hard problems into approximations with simpler primitives can be an effective tool to enable their use in practice. We plan to continue this challenge series that addresses critical challenges in human genome privacy protection.

## Additional file

Additional file 1: Figure S1. Timeline and statistics for the second Critical Assessment of Data Privacy and Protection (CADPP) workshop. (DOCX 982 kb)

## Abbreviations

GDS: NIH genome data sharing policy
HIPAA: Health Insurrance Portability and Accountability Act
HME: Homomorphic encryption
MAF: Minor allele frequency
PET: Privacy enhancing tehcnology
PGP: Personal genome project
SMC: Secure multiparty computing
VM: Virtual machine

## Footnotes

1
http://bmcmedinformdecismak.biomedcentral.com/articles/supplements/volume-15-supplement-5.
