# Supplementary material for: Protecting genomic data analytics in the cloud: state of the art and opportunities
Source: BMC Med Genomics. 2016 Oct 13;9:63. doi: 10.1186/s12920-016-0224-3 (PMC5062944; doi:10.1186/s12920-016-0224-3)
Supplement: Additional file 1: Figure S1. — Timeline and statistics for the second Critical Assessment of Data Privacy and Protection (CADPP) workshop. (DOCX 982 kb) [file 12920_2016_224_MOESM1_ESM.docx]

Protecting Genomic Data Analytics in the Cloud: State of the Art and Opportunities

Haixu Tang, Xiaoqian Jiang, Xiaofeng Wang, Shuang Wang, Heidi Sofia, Dov Fox

Kristin Lauter, Bradley Malin, Amalio Telenti, Li Xiong, Lucila Ohno-Machado

**Supplementary Figure 1:** Timeline and statistics for the second Critical Assessment of Data Privacy and Protection (CADPP) workshop
